# Homocysteine downregulates gene expression of heme oxygenase-1 in hepatocytes

**DOI:** 10.1186/1743-7075-11-55

**Published:** 2014-12-08

**Authors:** Xiaoqin Luo, Lei Xiao, Haixia Yang, Ruijuan Zhang, Manli Jiang, Jiahua Ni, Ting Lei, Nanping Wang

**Affiliations:** Cardiovascular Research Center, School of Medicine, Xi’an Jiaotong University, Xi’an, 710061 China; Department of Public Health, School of Medicine, Xi’an Jiaotong University, Xi’an, 710061 China; Nutrition and Food Safety Engineering Research Center of Shaanxi Province, School of Medicine, Xi’an Jiaotong University, Xi’an, 710061 China; Institute of Cardiovascular Science, Peking University, Beijing, 100191 China

**Keywords:** Homocysteine, Reactive oxygen species, Heme Oxygenase-1, Nrf2, Bach1

## Abstract

**Background:**

Hyperhomocysteinemia (HHcy) is an independent risk factor for liver diseases, such as fatty liver and hepatic fibrosis. However, the mechanisms underlying this pro-oxidative effect of homocysteine (Hcy) in hepatocytes remain largely unknown. Thus, we investigated the effect of Hcy on the gene expression of heme oxygenase-1 (HO-1), the primary rate-limiting enzyme in heme catabolism and a key anti-oxidant detoxification enzyme in maintaining cellular redox homeostasis.

**Methods:**

In vivo, twenty male C57BL/6 mice at 8 weeks of age were randomly divided into two groups. One group was fed a chow diet (chow group; n = 10), the other group of mice was fed a methionine-supplemented diet (Met group, 1 mg kg^−1^ day^−1^ L-methionine in drinking water; n = 10) for 4 weeks. In vitro, HepG2 cells were stimulated with different doses of homocysteine (Hcy).

**Results:**

Four weeks’ methionine supplementation caused a significant increase of plasma Hcy concentration and a decrease of HO-1 expression in the liver of C57BL/6 mice than mice received chow diet. Besides, SOD enzyme activities were impaired and the level of oxidative stress markers, such as malondialdehyde (MDA) were elevated in the liver from mice supplemented with methionine compared with control mice. In cultured hepatocytes, Hcy treatment reduced both the mRNA and protein levels of HO-1 dose-dependently. However, Hcy had no effect on the gene expression of Nrf2, the major transcriptional regulator of HO-1. Instead, Hcy induced the expression of Bach1, a transcriptional repressor of HO-1. In addition, Hcy stimulated the nuclear localization of Bach1 but prevented that of Nrf2. Furthermore, we found that knockdown of Bach1 attenuated the suppression of the HO-1 expression by Hcy.

**Conclusions:**

Collectively, our results demonstrated that Bach1 plays an important role in Hcy-triggered ROS generations through inhibiting HO-1 expression, likely, resulting from the disturbed interplay between Bach1 and Nrf2.

**Electronic supplementary material:**

The online version of this article (doi:10.1186/1743-7075-11-55) contains supplementary material, which is available to authorized users.

## Background

Homocysteine (Hcy) is a critical intermediate of methionine metabolism and has profound importance in health and diseases [1]. Indeed, the metabolism of Hcy mainly takes place in liver and normal concentrations of total Hcy in human plasma are 5–16 μmol/L [2]. The disrupted metabolism of Hcy causes hyperhomocysteinemia (HHcy). There are three ranges of HHcy: moderate (16–30 μmol/L), intermediate (31–100 μmol/L), and severe (>100 μmol/L) [3]. Epidemiological studies have demonstrated that HHcy is an independent risk factor for fatty liver diseases, cardiovascular disease, diabetes and so on [3–7]. Clinical and experimental studies have shown that pathophysiological effects of Hcy involved the excessive generation of reactive oxygen species (ROS) [8–10] *.* However, the molecular mechanisms underlying the effect of Hcy on oxidative stress have not been explored.

Heme oxygenase-1 (HO-1), the primary rate-limiting enzyme in heme catabolism, acts as a key anti-oxidant detoxification enzyme in maintaining cellular redox homeostasis [11]. HO-1 is involved in many pathophysiological changes of liver injuries including liver oxidative stress, chronic inflammation and so on. It has been reported that the induction of HO-1 as well as its reaction products can protect the liver against damage caused by a number of chemical compounds [12–16]. Otherwise, HO-1 knockout mice developed major hemosiderosis and chronic inflammatory in liver, suggesting HO-1 induction as an important cellular endeavor for hepatoprotection [17, 18]. The primary mechanism for up-regulation of the HO-1 gene is by increasing transcription of this gene [19, 20]. This process is mediated by binding of nuclear factor erythroid 2-related factor 2 (Nrf2), a basic leucine zipper transcription factor, to antioxidant response element (ARE) sequence [21]. In the presence of oxidative stimuli, activated Nrf2 translocates into the nucleus, binds to the heme-responsive elements (HeRE) in the 5′-UTR of the HO-1 promoter, and commences transcription of HO-1 [22, 23]. However, the regulation of HO-1 in HHcy has not been well studied. Therefore, we examined how Hcy modulates the stress protein HO-1 in hepatocytes. In the current study, we demonstrated that Nrf2 as well as Bach1 are involved in the downregulation of HO-1 gene expression by Hcy. The subcellular localisation of these two transcription factors plays critical roles in this regulating effect.

## Methods

### Animal experiments

The animal experiments conform to the Guide for the Care and Use of Laboratory Animals that was published by the US National Institute of Health (NIH Publication No. 8523, revised 1985). Male C57BL/6 mice at 8 weeks of age were obtained from the Experimental Animal Center of Xi’an Jiaotong University (Xi’an, China) and fed with either chow diet (n = 10) or methionine-rich diet (1 mg kg^−1^ day^−1^ L-methionine in drinking water; n = 10) for 4 weeks. Each mouse was separately housed in a temperature-controlled (24°C) facility with a 12 h light/12 h dark cycle with free access to food and autoclaved water. We weighed the mice every week and calculated the consumption of water and food for each group. After being anaesthetized by intraperitoneal injection of 3% chloral hydrate, the blood of mice were collected from the orbit. The study and all of the procedures were approved by the Xi’an Jiaotong University Animal Experiment Committee.

### Plasma Hcy determination

Plasma Hcy concentrations were measured as previously described [24]. Briefly, the blood samples were collected in EDTA-containing tubes, which were then immediately centrifuged at 3,000 g for 10 min. The concentrations of plasma total Hcy concentrations were quantified using a fluorescence polarization immunoassay ELISA Kit (Abbott IMx).

### SOD enzyme activity unit determination

The livers were blotted dry and weighed and then homogenized in 8 volumes of lysis buffer (pH7.4) at 4°C for 30 seconds using a polytron homogenizer. The homogenate was centrifuged at 12,000 × g for 15 min, and then was used to detect the activity units of SOD enzyme by a commercial kit (Biovision).

### Determination of Malondialdehyde (MDA)

The protein concentration of the supernatants of liver homogenate was determined using the Pierce™ BCA Protein assay kit (Thermo Fisher Scientific Inc., Rockford, USA) according to the user manual. The MDA concentrations were detected with a kit according to the manufacturer’s instructions (R&D Systems, Inc., USA) and normalized to protein levels.

### Cell culture

HepG2 cells (ATCC, Manassas, VA, USA) were cultured with Dulbecco’s Modified Eagle’s Medium (DMEM) containing 10% fetal bovine serum (FBS). Cells were seeded into 6-well plates 24 h prior to treatments at approximately 80% confluence and exposed to different doses of Hcy (Sigma-Aldrich, St. Louis, MO) for additional 24 h.

### Quantitative reverse transcription PCR (qRT-PCR)

The total RNA of liver homogenate and cells were obtained and qRT-PCR was conducted as previously described [25]. Primers against Bach1 (forward primer, 5′-GGACACTCCTTGCCAAATGCAG-3′; reverse primer, 5′-TGACCTGGTTCTGGGCTCTCAC-3′); Nrf2 (forward primer, 5′-AGC ACACCCAGTCAGAAACCAG-3′; reverse primer, 5′-TCTACAAACGGGAATGTCG-3′); HO-1 (forward primer, 5′-CGCTGGCAGGAGGTCAT-3′; reverse primer, 5′-CATCGGAGAAGCGGAGC-3′) and GAPDH ((forward primer, 5′- ACCACAGTCCATGCCATCAC-3′; reverse primer, 5′-TCCACCACCCTGTTGCTGTA-3′) were designed using the sequence information of the NCBI database. The PCR conditions were as follows: initial denaturation: 95°C, 5 min; 40 cycles of denaturation (95°C, 30 s), annealing (58°C, 30 s), and elongation (72°C, 60 s). The fluorescent signals were collected during the extension phase, Ct values of the sample were calculated, and the mRNA levels were analyzed by 2^-△△Ct^ method.

### Western blotting

Proteins from liver homogenate were extracted with ice-cold lysis buffer (Roche, Mannheim, Germany). Additionally, the nuclear proteins were extracted by the Pierce NE-PER kit (Pierce, Rockford, IL, USA) according to the manufacturer’s instruction. All steps were carried out on ice. Proteins concentrations were determined by the BCA assay (Pierce). Protein (25 μg) from each sample was separated by 10–12% SDS-PAGE and electrotransferred to Polyvinylidene Fluoride (PVDF) membranes. The membranes were blocked with 5% BSA for 1 h at room temperature and incubated overnight at 4°C using the following 1:1000 primary rabbit antibodies (Cell Signaling Technology Inc. MA, USA) of HO-1 (#5853), Bach1 (#4578), Nrf2 (#12721), Histone and mouse antibody of β-actin, followed by 1:4000 dilution of goat anti-rabbit or goat anti-mouse horseradish peroxidase-labeled antibodies (Cell Signaling Technology Inc. MA, USA). The bands were visualized using the ECL system, and the band density was determined by Image J software (NIH, USA).

### Measurement of reactive oxygen species

The intracellular ROS level of cells was detected by fluorescence microscope and flow cytometry using the fluorescent probe 2′,7′-Dichlorofluorescin diacetate (DCFH-DA), which is a cell-permeable non-fluorescent probe and turns to highly fluorescent 2′,7′-Dichlorofluorescin (DCF) upon oxidation. The cells were quiesced for 24 h and treated with different doses of Hcy for additional 24 h or H_2_O_2_ (100 μM) for 30 min as a positive control. Then, the cells were washed with PBS, and incubated with 10 μM DCFH-DA at 37°C for 30 min in dark. After incubation, the cells were washed with PBS and observed under fluorescent microscopy directly or adjusted to a concentration of 1 × 10^6^/mL after trypsinization and analyzed by FACScan (BD FACSAriaTM III, United States) within 30 min. 10,000 cells were counted in each determination and the specific fluorescence signals were collected with a 488-nm band pass filter.

### Luciferase reporter assay

The HO-1 promoter reporter (pGL3-HO-1) was constructed. The fragment spanning from −3000 base pairs upstream of the transcription start of the human HO-1 gene was subcloned into pGL3-basic plasmid containing the ARE sequence and the firefly luciferase reporter gene (Promega Corporation, Madison, USA). The reporter plasmid and β-gal were transfected into HepG2 cells using the Lipofectamine 2000 reagents from Invitrogen according to the manufacturer’s instructions. These cells were subsequently treated with different dose of Hcy (0, 100, 500, 1000 μM) for 24 h. Cell lysates were harvested to measure the luciferase activities using the Dual-Luciferase® Reporter Assay System (Promega). The luciferase activities were then normalized to β-galactosidase activity.

### Gene silencing

To knock down Bach1 expression, cells were transfected with 100 pmol Bach1 siRNA, a commercially available small interfering RNAs (siRNAs) duplex components against this molecule (sc-37064; Santa Cruz, CA, USA) or a negative control siRNA (sc-37007; Santa Cruz, CA, USA), a RNA duplex with no known sequence homology, in a 12-well format using Lipofectamine 2000 (Invitrogen, Carlsbad, CA) according to the manufacturer’s instructions.

### Statistical analysis

All of the experiments were repeated at least three times. Data are expressed as means ± SEM. The significance of differences between 2 or more than 2 groups was determined by Tamhane’s test and one-way ANOVA, respectively, using SPSS 16.0 software (SPSS, Chicago, IL). A value of *P* < 0.05 was considered to be statistically significant.

## Results

### Hcy accumulation increased oxidative stress and down-regulated HO-1 expression in mouse livers

**Figure 1:**
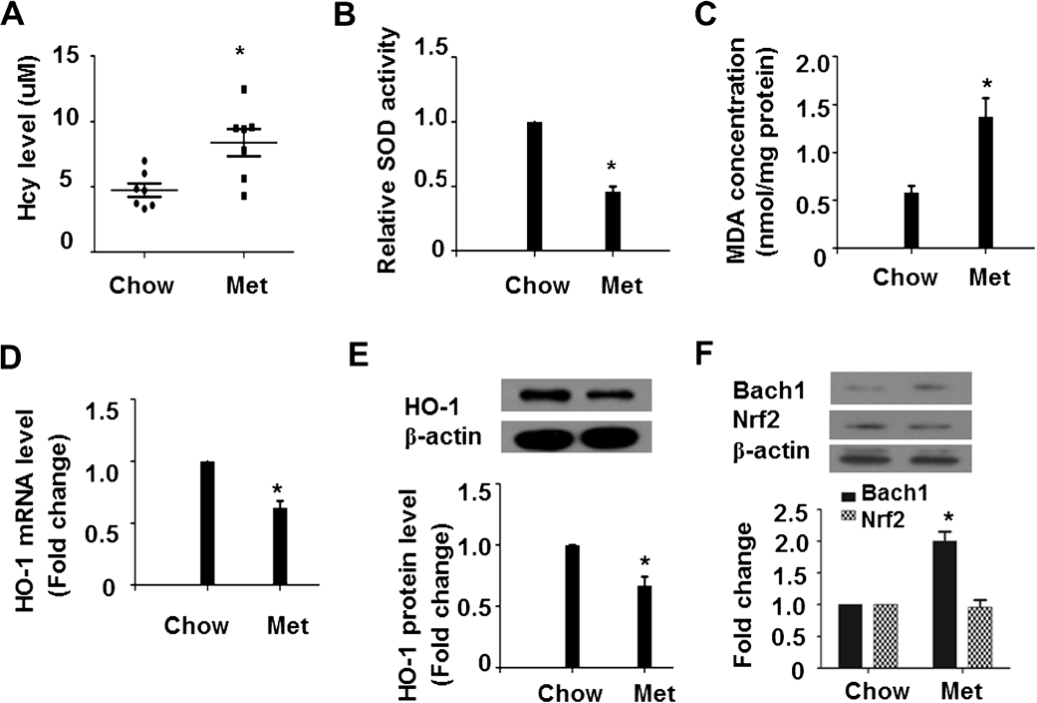
**Hcy accumulation resulted in oxidative stress and HO-1 downregulation. (A)** Hcy concentrations of plasma were determined by an ELISA assay and indicated for each group of C57BL/6 J mice (n = 7 for each group). The livers were homogenized and the total protein concentrations were assessed to determine the enzyme activity units of SOD by WST-1 method **(B)** and the MDA concentrations using an ELISA assay **(C)**. **(D)** The total RNA of livers were extracted and subjected to qRT-PCR for the assessment of HO-1 mRNA level. The bar graph shows mRNA levels of HO-1 after normalization to GAPDH. Protein levels of HO-1, Bach1 and Nrf2 were analyzed using Western blotting **(E and F)**. β-actin was used as an internal control to ensure equal loading in all lanes of the gel. Representative results of 3 independent experiments are shown. Data are presented as means ± SEM from 3 three independent experiments. *P < 0.05 vs. mice fed with chow diet.

To determine the effect of Hcy on HO-1 expression, mice were supplemented with L-methionine in drinking water (Met group). After 4 weeks of treatment, the plasma Hcy concentrations significantly raised compared to those in chow diet-fed mice (chow group) (Figure 1A). It is well established that oxidative stress is implicated in Hcy-caused liver injury. Relative analysis demonstrated that SOD enzyme activity was decreased significantly to 45.9% in Met groups compared with the Chow group mice (Figure 1B). Furthermore, the mice from Met group had higher levels of MDA in livers than chow group (Figure 1C). Next, we identify the effect of elevated Hcy on the expression of HO-1, a key anti-oxidant detoxification enzyme in maintaining cellular redox homeostasis. The mRNA levels of HO-1 were remarkably reduced in Met group than chow group (Figure 1D). Then, liver homogenate supernatants were then assayed by Western blotting. Consistently, the mice from Met group had an obvious decrease in HO-1 expression compared with chow group (Figure 1E). We also detected the effects of increased level of Hcy on the expression of Bach1 and Nrf2, which are two main regulator of HO-1. As shown in Figure 1F, Met supplementation induced Bach1 protein level whereas the expression of Nrf2 was comparable between two groups.

### Hcy dose-dependently induced intracellular ROS production in hepatocytes

**Figure 2:**
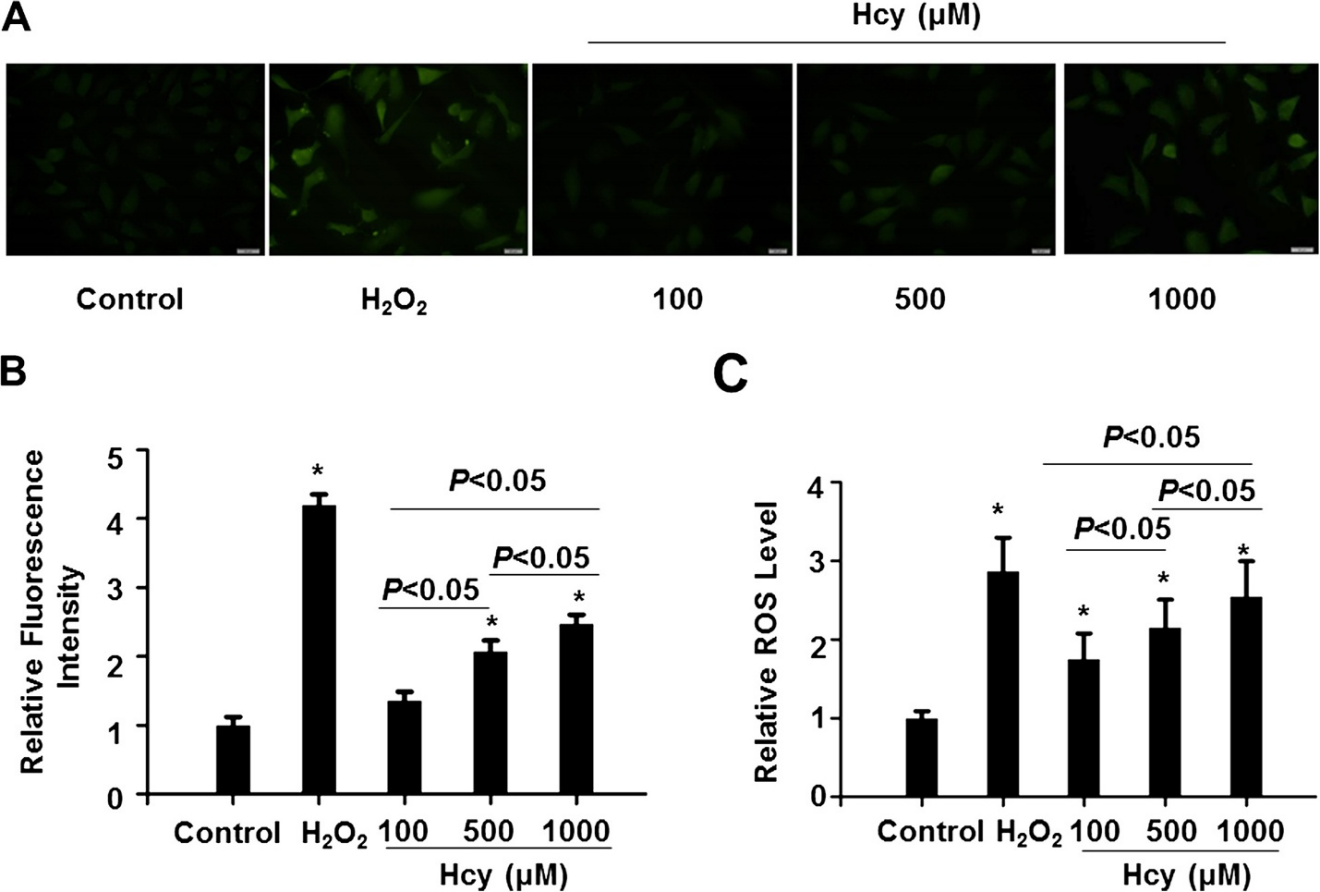
**Effect of Hcy on intracellular ROS production.** Cells were cultured without serum for 24 h and then treated with Hcy (100, 500, 1000 μM) for another 24 h. The intracellular ROS level of cells was detected by fluorescence microscope and flow cytometry using the fluorescent probe DCFH-DA (10 μM, 30 min). H_2_O_2_ (100 μM, 30 min) treatment is a positive control. **(A)** The ROS production was detected by DCFH-DA staining. The images are representative of typical staining (400×; scale bar: 20 μM). Digital scans of DCHF-DA-stained cells were quantified using Image J software **(B)**. Data are shown as means ± SEM of three independent experiments. **(C)** The ROS levels were detected by flow cytometry analysis. Data are represented as percentages of the control group from six independent experiments (*P < 0.05 vs. control).

Next, we performed in vitro culture of HepG2 cells with Hcy. The concentrations in this study did not affect cell viability (Additional file 1: Figure S1). The effect of Hcy on intracellular ROS levels is shown in Figure 2. Cells were treated with different doses of Hcy for 24 h and the ROS levels were determined by flow cytometry or fluorescent microscope after incubation of the fluorescent probe DCFH-DA. Hcy increased the intracellular ROS levels in a dose-dependent manner (Figure 2A and 2B). As a positive control, the treatment with 100 μM H_2_O_2_ for 30 min significantly triggered ROS generation of HepG2 cells. Similar results were obtained by fluorescent microscope. Hcy dose-dependently enhanced the intensity of fluorescence signal of DCF (Figure 2C).

### Hcy down-regulates HO-1 expression

**Figure 3:**
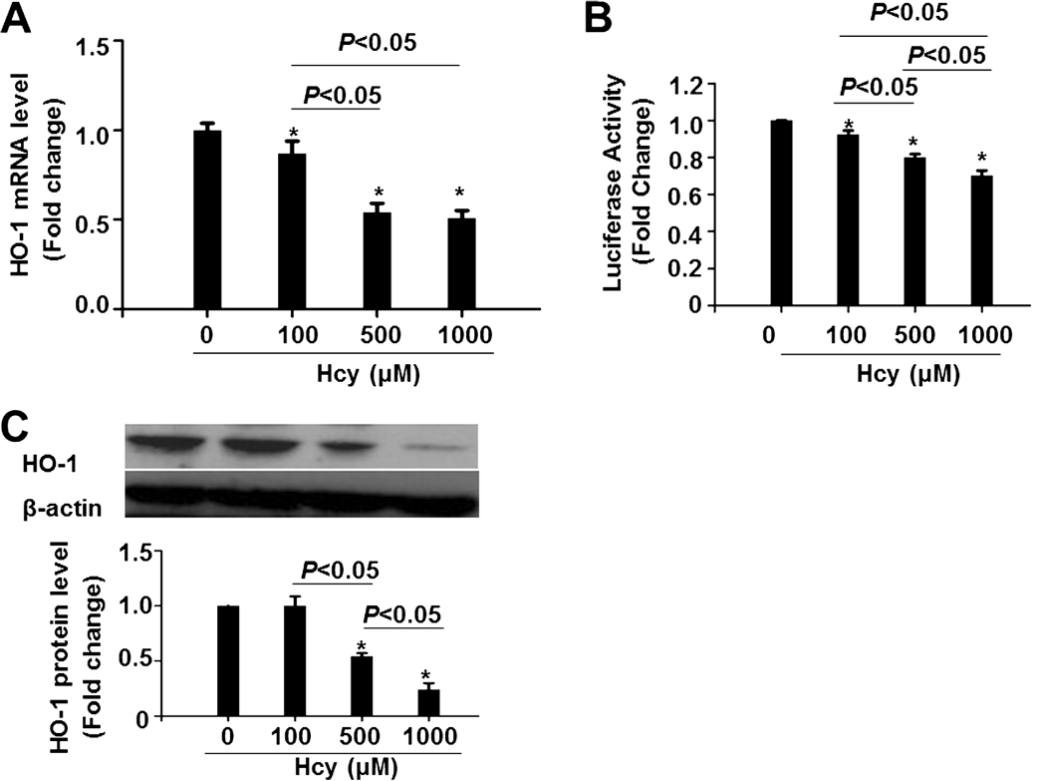
**Hcy dose-dependently down-regulates HO-1 gene expression in HepG2 cells.** HepG2 cells were treated with the indicated concentrations of Hcy for 24 h. **(A)** Total RNA was extracted and subjected to qRT-PCR for the assessment of HO-1 mRNA level. The bar graph shows mRNA levels of HO-1 after normalization to GAPDH. Data are presented as means ± SEM from 3 three independent experiments. *P < 0.05 vs. control. **(B)** HepG2 cells were transfected with the HO-1-pGL3 plasmid and then exposed to Hcy for 24 h. The data are expressed as fold change of the luciferase activities normalized toβ-gal activity compared to control. *P < 0.05 vs. control; n = 4. **(C)** Protein levels of HO-1 were analyzed using Western blotting. Representative results of three independent experiments are shown. β-actin was used as an internal control to ensure equal loading in all lanes of the gel.

To determine the effect of Hcy on HO-1 expression in HepG2 cells, qRT-PCR and Western blotting were performed to detect the mRNA and protein levels of HO-1, respectively. As shown in Figure 3A, Hcy dose-dependently decreased the HO-1 mRNA levels after 24 h exposure. The statistical analysis showed that HO-1 expression levels significantly differ from each other (*P* < 0.05). In order to examine whether the effect of Hcy on HO-1 mRNA expression is on a transcriptional level, we cloned a luciferase-reporter driven by the 3 kb HO-1 promoter fragment of the human HO-1 gene. The results of luciferase assays showed that Hcy inactivated the HO-1 promoter dose-dependently (Figure 3B). Next, we detected the expression of HO-1 protein by western blotting assays. Compared with untreated cells, HO-1 protein expression declined to 98.1%, 54.3% and 23.7% in cells treated with Hcy at the concentrations of 100, 500 and 1000 μM, respectively, and the differences were statistically significant from each other (Figure 3C). We also determined the mRNA level of Nqo1 and GSTA1, other two target genes of Nrf2, and found that Hcy reduced the expression of Nqo1 dose-dependently whereas the expression of GSTA1 was only decreased in higher doses of Hcy (Additional file 1: Figure S2).

### Hcy had opposite effects on the nuclear localization of Bach1 and Nrf2

**Figure 4:**
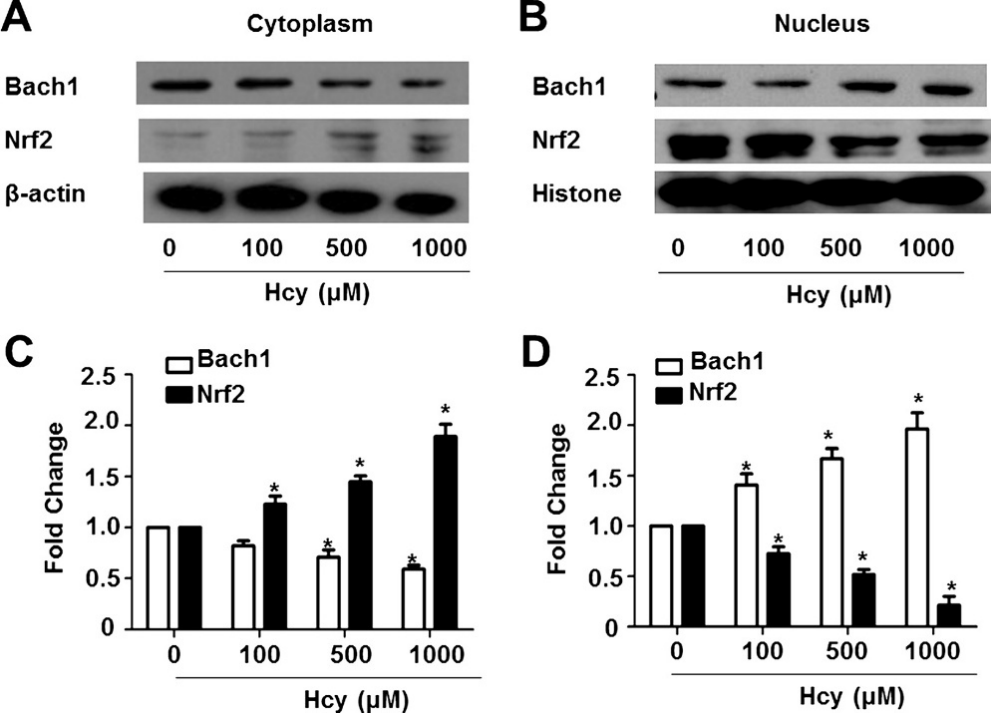
**Hcy induced nuclear import of Bach1 and the export of Nrf2.** The cytosolic and nuclear proteins were extracted separately after Hcy treatment and subjected to Western blotting assay. Representative images of three independent experiments were shown. The cytosolic **(A)** and nuclear **(B)** proteins were present to illustrate the changes of subcellular localization of Nrf2 and Bach1. The values of density of proteins were justified with β-actin **(C)** and Histone **(D)**, accordingly. The relative density ratios of untreated cells were set at a value of 1.0. The values are means ± SEM from three independent experiments. *P < 0.05 vs. control.

Since Bach1 can down-regulate HO-1 transcription through competing with Nrf2 for the binding of antioxidant response element (ARE), the levels of nuclear and cytosolic Bach1 and Nrf2 were examined by Western blotting. The proteins in cytoplasm were shown in Figure 4A, the Nrf2 protein elevated gradually following Hcy treatment at concentrations of 100, 500 and 1000 μM while the fractions of Bach1 were decreased remarkably compared to those of control cells. The statistical analysis showed significant differences from each other (Figure 4C, *P* < 0.05). In contrast with the localization of the two proteins in cytoplasm, nuclear Bach1 accumulated and nuclear Nrf2 reduced dramatically after Hcy exposure at the concentrations above (Figure 4B and D).

### Hcy induced the expression of Bach1 but not Nrf2 in HepG2 cells

**Figure 5:**
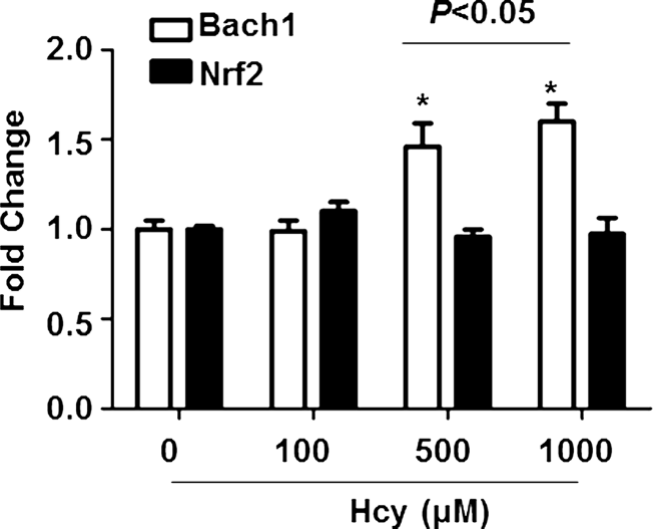
**Hcy dose-dependently increases Bach1 but not Nrf2 mRNA levels.** Cells were treated with Hcy at indicated concentrations for 24 h. The mRNA levels of Bach1 and Nrf2 were quantified by qRT-PCR assay. Relative mRNA amounts of Bach1 and Nrf2 were normalized to GAPDH. Data shown are as means ± SEM of three independent experiments. *P < 0.05 vs. control.

We next evaluate the effect of Hcy on Nrf2 gene expression since Nrf2 is involved in cellular protection against oxidative stress through antioxidant response element (ARE)-directed induction of several phase 2 detoxifying and antioxidant enzymes, including HO-1 [26]. Interestingly, Hcy had no significant effect on Nrf2 mRNA levels. In contrast, the mRNA level of another transcription factor Bach1, which was suggested to represses the transcription of HO-1, increased in a concentration-related manner after Hcy treatment (Figure 5).

### Bach1 siRNA reversed the suppression of HO-1 expression by Hcy

**Figure 6:**
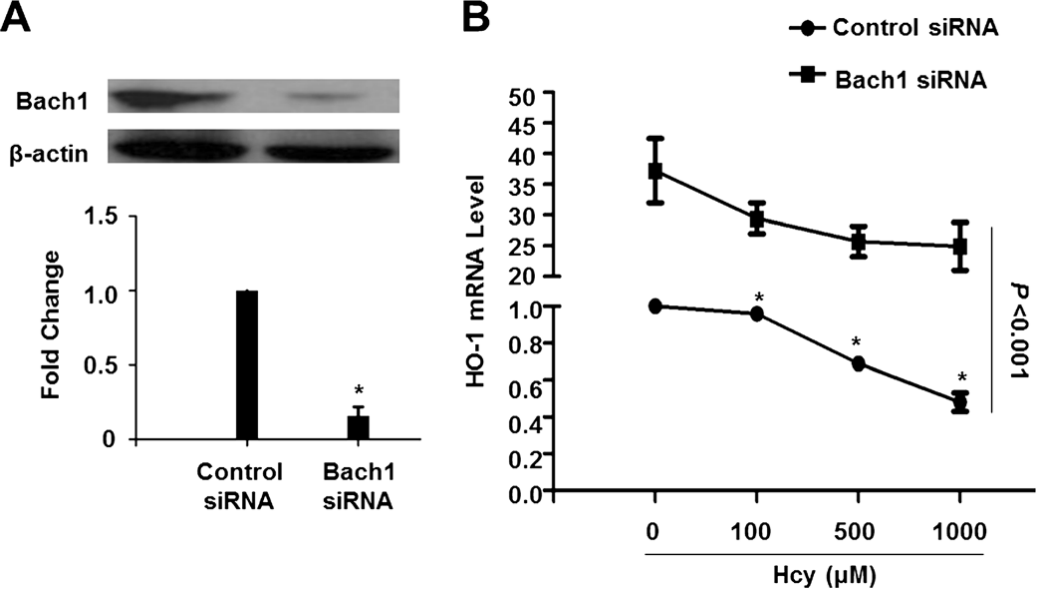
**The effect of Bach1 on the regulation of HO-1 expression in Hcy-treated HepG2 cells.** Cells were treated with 100 pmol Bach1siRNA or control siRNA for 72 h before Hcy treatment (0, 100, 500, 1000 μM for 24 h). **(A)** Protein level of Bach1 was analyzed using Western blotting. β-actin was used as a internal control. Representative image of three independent experiments was shown. **(B)** HO-1 mRNA levels were detected by qRT-PCR. Each data point represents means ± SEM from three independent experiments. Values for cells treated with control siRNA-only were set equal to 1. The two curves are significantly different from one another using nonlinear regression analysis (*P* < 0.001) and the data points are significantly different from one another. *P < 0.05 vs. cells treated with control siRNA-only.

To determine the effect of Bach1 on the regulation of HO-1 expression in Hcy-treated HepG2 cells, we silenced human *Bach1* gene expression by Bach1 siRNA. After the transfection of 100 pmol Bach1 siRNA for 72 h, the Bach1 protein level was markedly reduced to 16% compared to those of control siRNA-treated cells (Figure 6A). The levels of HO-1 mRNA were significantly increased (37.1-fold) after exposure to Bach1 siRNA without Hcy treatment. Compared to control siRNA-treated cells, Bach1 siRNA treatment significantly reversed the suppression of HO-1 gene expression by Hcy (Figure 6B). However, Hcy failed to attenuate the expression of HO-1 significantly in Bach1 siRNA-treated cells even with a slight decrease (P = 0.12, 0.09 and 0.07 for Hcy at the concentrations of 100, 500 and 1000 μM, respectively, when compared with Bach1 siRNA-treated cells only).

## Discussion

Hyperhomocysteinemia (HHcy) is an independent risk factor for liver diseases, such as fatty liver and hepatic fibrosis [5]. The pathophysiological changes of HHcy-induced liver injuries include chronic inflammation, fibrosis, cirrhosis, and so on. In the present study, we provided novel result that Hcy decreased the expression of HO-1, a key anti-oxidant detoxification enzyme, via deregulated interplay between Nrf2 and Bach1, resulting oxidative stress in hepatocytes. This effect of Hcy on HO-1 is highly dose-dependent and may represent another mechanism contributing to the pro-oxidative effects of Hcy.

Nutritional factors such as high methionine-diet are associated with increased serum homocysteine levels. Extensive studies have shown that a high methionine-diet had awful effects on liver pathophysiology, for instance, oxidative stress, inflammation, lipid metabolism disorders and fibrosis [27, 28]. Our observations are in line with previous studies reporting that methionine supplementation increased plasma Hcy levels, triggered oxidative stress of livers and impaired the anti-oxidative enzyme activity. Furthermore, methionine treatment attenuated the expression of HO-1 in the liver. Therefore, we next performed in vitro culture of hepatocytes to Hcy and found that Hcy treatment dose-dependently down-regulates HO-1 expression. These results suggested that Hcy accumulation has a promoting effect on oxidative stress, which might be partially contributed by the decrease of anti-oxidative enzyme HO-1. However, whether Hcy affect the HO-1 enzymatic activity remains to be characterized and this limitation is warranted to be examined in future study.

Nrf2 is the main transcriptional activator of the HO-1 gene in response to various forms of stimulation such as oxidative stress [29]. Activation of the Nrf2/HO-1 pathway can protect cells from oxidative stress-induced damage [30]. Antioxidants are strong activators of Nrf2 because after metabolism they produce a small amount of oxidative stress that signals Nrf2 activation [31–33]. Inversely, chemicals with oxidant stimulation are able to repress Nrf2/HO-1 pathway [34–36]. In the present study, we found that Hcy which triggered ROS generation significantly inhibited HO-1 mRNA and protein levels in HepG2 cells. As for the result that Nrf2 mRNA levels did not change obviously after Hcy treatment, it is possible that a decreased binding of Nrf2 to ARE sequence is a potential mechanism as well. Firstly, the decreased nuclear translocation of Nrf2 would lead to a reduced binding to its cognate sequences in the target gene HO-1. Secondly, the transfection and ChIP assays indeed confirmed that Bach1 and Nrf2 competed with each other on the ARE sequence in HepG2 cells [37].

Like Nrf2, Bach1 is also a member of the basic leucine zipper family of proteins [38]. It was previously reported that Bach1 plays a critical role in heme-dependent down-regulation of the human HO-1 gene [39, 40]. Sun *et al.* have showed that HO-1 is constitutively expressed at higher levels in many tissues of Bach1-deficient mice, suggesting that Bach-1 acts as a negative regulator of transcription of the mouse *HO-1* gene [41]. Here, our results demonstrated that Hcy induced Bach1 expression at the transcriptional level. Further RNA interference experiments allowed us to confirm the core role of Bach1 in the suppression of HO-1 to Hcy. On the other hand, Bach1 can regulate HO-1 gene expression by competing with Nrf2 [42, 43]. Under oxidative stress, Nrf2 is released from Keap1 and translocated into the nucleus. In the nucleus, Bach1 can form heterodimers with the Maf-related protein family [44], and then leads to switching off Nrf2 and suppression of cytoprotective gene expression to basal levels through competition with Nrf2 for binding to ARE [37]. Hence, the nuclear import of Bach1 is supposed to allow for unhindered nuclear export of Nrf2. As expected, we found Hcy treatment caused an alteration of nuclear localizations of Bach1 and Nrf2. Consequently, this modification process of the two transcription factors was associated with a significant decrease in the expression of the downstream cytoprotective phase 2 genes HO-1. These results demonstrated that the increase of Bach1 gene expression and its nuclear translocation potentiated the down-regulation of the HO-1 by Hcy.

A previous study has suggested that incubation of Hcy for very short time (45 min) induced the expression of HO-1 in HepG2 cells [45]. It seems that the oxidative stress provoked by Hcy stimulated the immediate cellular response in HepG2 cells by activating the Nrf2-ARE pathway. However, some certain degree of maladaptation will occur when cells were exposed to higher levels of Hcy at longer duration. It has been reported that high levels of Hcy (500 μM) were able to abolish hypoxia-mediated HO-1 expression in a concentration-dependent manner [46]. Actually, the mechanism of signal transduction from antioxidant to Nrf2 is complex and involves basal, pre-induction, induction and post-induction phases [47]. Cellular exposure to oxidants usually firstly triggers the pre-induction response in which negative regulators of Nrf2 are exported out of nucleus. Consequently, Nrf2 itself is activated and increase HO-1 transcription to defense oxidative stress. However, it is worth noting that this protective benefit is limited since the induction phase Nrf2 is followed by the post-induction phase, which switches off Nrf2 activation [48].

**Figure 7:**
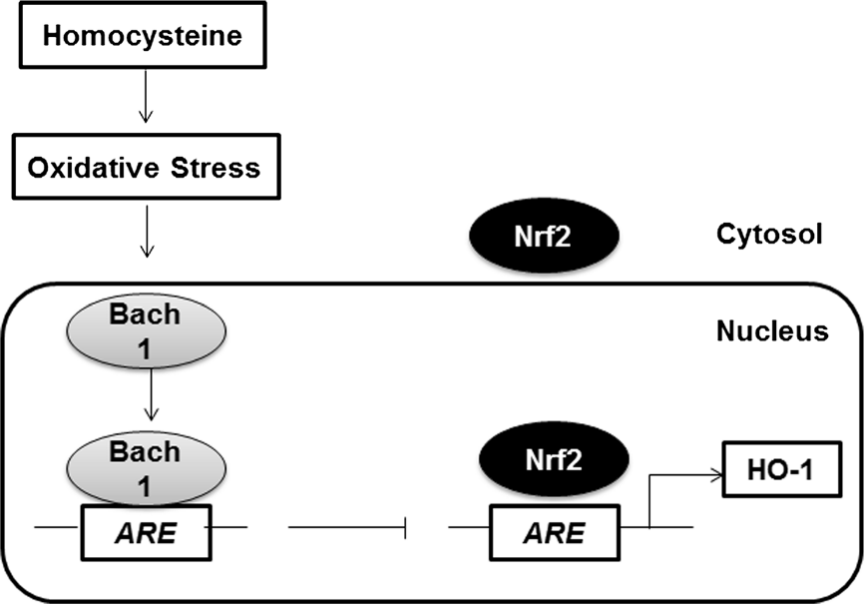
**Schematic model of effect of Hcy on HO-1 expression via regulation of Bach1 and Nrf2.** With high levels of Hcy, expression of Bach1, a repressor of HO-1 gene expression, is increased. Bach1 also competes with Nrf2 to bind antioxidant response element (ARE), then reduces HO-1 transcription.

In summary, our results showed that Hcy markedly reduces HO-1 mRNA and protein levels in hepatocytes. This down-regulation effect was associated with up-regulation of Bach1 mRNA levels. In addition, Hcy promoted the alteration of nuclear localization of Bach1and Nrf2 (Figure 7). The results of our study confirmed that the subcelluar localizations of Nrf2 and Bach1 caused by the excessive oxidative stress in Hcy-treated hepatocytes could account for the down regulation of HO-1 expression, which may be responsive for the HHcy-induced liver injury.

## Electronic supplementary material

Additional file 1: Figure S1: The effect of different dose of Hcy on cell viability. After the treatment of different doses of Hcy for 24 h, cells were incubated for additional 4 h in the presence of a MTT labelling mixture. The absorbance of the samples was measured at 550 nm with using a microliter plate (ELISA) reader. The results were presented as fold change of the absorbance against the blank compared to control. **Figure S2.** Hcy down-regulates gene expressions of Nqo1 and GSTA1. HepG2 cells were treated with the indicated concentrations of Hcy for 24 h. Total RNA was extracted and subjected to qRT-PCR for the assessment of Nqo1 (A) and GSTA1 (B) mRNA levels. The bar graph shows mRNA levels of the two genes after normalization to GAPDH. Data are presented as means ± SEM from 3 three independent experiments. *P < 0.05 vs. control. (DOCX 207 KB)

## Abbreviations

Hcy: Homocysteine
ROS: Reactive oxygen species
HO-1: Heme oxygenase-1
Nrf2: Nuclear factor erythroid 2-related factor 2
ARE: Antioxidant response element
siRNA: Small interfering RNA
GAPDH: Glyceraldehyde-3-phosphate dehydrogenase
DCFH-DA: 2′,7′-Dichlorofluorescin diacetate
PVDF: Polyvinylidene fluoride.
