# Supplementary material for: Homocysteine downregulates gene expression of heme oxygenase-1 in hepatocytes
Source: Nutr Metab (Lond). 2014 Dec 8;11:55. doi: 10.1186/1743-7075-11-55 (PMC4268895; doi:10.1186/1743-7075-11-55)
Supplement: Supplementary file 1 — Additional file 1: Figure S1: The effect of different dose of Hcy on cell viability. After the treatment of different doses of Hcy for 24 h, cells were incubated for additional 4 h in the presence of a MTT labelling mixture. The absorbance of the samples was measured at 550 nm with using a microliter plate (ELISA) reader. The results were presented as fold change of the absorbance against the blank compared to control. Figure S2. Hcy down-regulates gene expressions of Nqo1 and GSTA1. HepG2 cells were treated with the indicated concentrations of Hcy for 24 h. Total RNA was extracted and subjected to qRT-PCR for the assessment of Nqo1 (A) and GSTA1 (B) mRNA levels. The bar graph shows mRNA levels of the two genes after normalization to GAPDH. Data are presented as means ± SEM from 3 three independent experiments. *P < 0.05 vs. control. (DOCX 207 KB) [file 12986_2014_625_MOESM1_ESM.docx]

**Additional file 1**

**MS No.: 2104791932137059**

**MS TITLE: Homocysteine Downregulates Gene Expression of Heme Oxygenase-1 in Hepatocytes**

**Methods**

**Cell viability test**

HepG2 cells were cultured with DMEM containing 10% FBS. Cells were seeded into 96-well plates 24 h prior to treatments at approximately 80% confluence. Cell viability was then determined by a MTT assay. Briefly, after the exposure to different doses of Hcy for 24 h, cells were incubated for additional 4 h in the presence of a MTT labelling mixture (working concentration: 0.5 mg/ml). The absorbance of the samples was measured at 550 nm with using a microliter plate (ELISA) reader (Molecular Devices, Sunnyvale, CA, USA) against a background control (medium alone) as a blank.

**Quantitative reverse transcription PCR (qRT-PCR)**

The total RNA of cells was obtained and qRT-PCR was conducted regularly. Primers against human Nqo1 (forward primer, 5’-GGAAGCTGCAGACCTGGTGA-3’; reverse primer, 5’-CCTTTCAGAATGGCTGGCA-3’); human **GSTA1** (forward primer, 5’-CCCCTTTCCCTCTGCTGAAG-3’; reverse primer, 5’-TGCAGCTTCACTGAATCTTGAAAG-3’) and GAPDH ((forward primer, 5’-ACCACAGTCCATGCCATCAC-3’; reverse primer, 5’-TCCACCACCCTGTTGCTGTA-3’) were designed using the sequence information of the NCBI database. The fluorescent signals were collected during the extension phase, Ct values of the sample were calculated, and mRNA levels were analyzed by 2^-ΔΔCt^ method.

**Supplemental figures**

**Additional file 1: Figure S1**


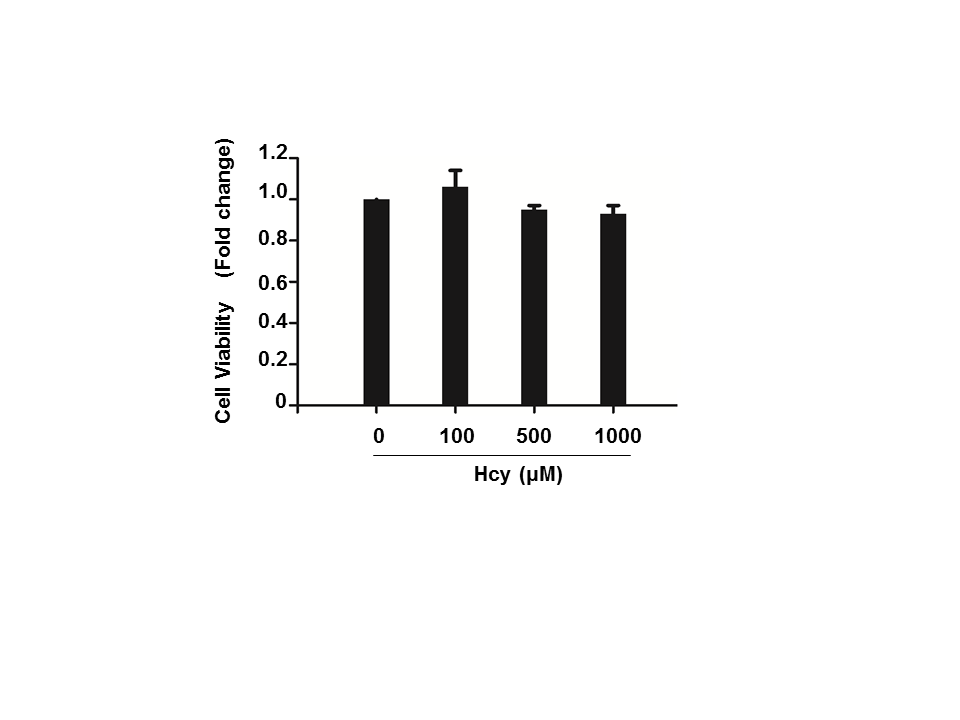


**Figure S1 The effect of different dose of Hcy on cell viability.**

After the treatment of different doses of Hcy for 24 h, cells were incubated for additional 4 h in the presence of a MTT labelling mixture. The absorbance of the samples was measured at 550 nm with using a microliter plate (ELISA) reader. The results were presented as fold change of the absorbance against the blank compared to control.

**Additional file 1: Figure S2**


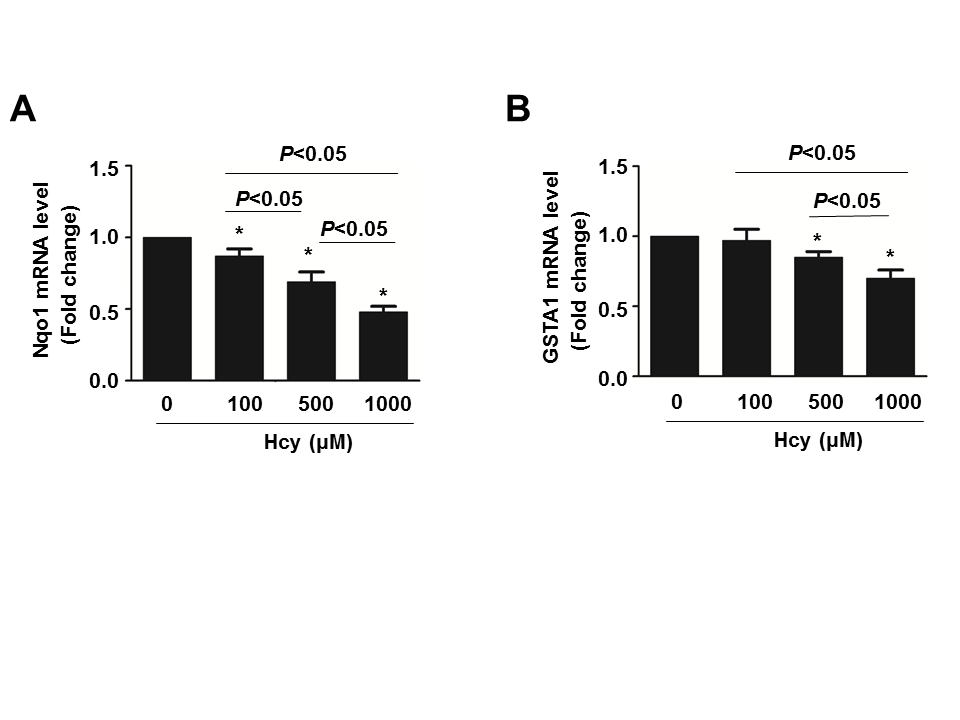


**Figure S2 Hcy down-regulates gene expressions of Nqo1 and GSTA1.**

HepG2 cells were treated with the indicated concentrations of Hcy for 24 h. Total RNA was extracted and subjected to qRT-PCR for the assessment of Nqo1 (A) and GSTA1 (B) mRNA levels. The bar graph shows mRNA levels of the two genes after normalization to GAPDH. Data are presented as means ± SEM from 3 three independent experiments. **P* < 0.05 vs. control.
